# Exploring the perioperative experiences of youth undergoing cardiac surgery: A qualitative needs assessment for tailoring a mobile self-management app

**DOI:** 10.1017/cts.2026.10741

**Published:** 2026-04-22

**Authors:** Tieghan Killackey, Jill O’Hare, Sandra Merklinger, Lauren Harris, Mansi Patel, Nicole Drumm, Selini Jayawickrema, Fiona Campbell, Katherine Taylor, Jennifer Russell, Rachel D. Vanderlaan, Fareha Nishat, Laura Veloso, Maya Samarasekera, Jennifer N. Stinson

**Affiliations:** 1 Peter Munk Cardiac Centre, University Health Network, Toronto, ON, Canada; 2 Lawrence Bloomberg Faculty of Nursing, University of Toronto, Toronto, ON, Canada; 3 Labatt Family Heart Centre, The Hospital for Sick Children, Toronto, ON, Canada; 4 Child Health Evaluative Sciences, Hospital for Sick Children Research Institute, Toronto, ON, Canada; 5 School of Nursing, York University, North York, ON, Canada; 6 Department of Critical Care, William Osler Health System, Brampton, ON, Canada; 7 Department of Anesthesiology and Pain Medicine, University of Toronto, Toronto, ON, Canada; 8 Department of Anesthesia and Pain Medicine, The Hospital for Sick Children, Toronto, ON, Canada; 9 Parent partner, Canada; 10 Youth Partner, Canada

**Keywords:** Postoperative pain, cardiovascular surgery, mobile applications, self-management, adolescent

## Abstract

**Introduction:**

One in five children who undergo cardiac surgery report experiencing moderate to severe pain lasting more than 3 months after surgery which has a significant impact on quality of life. iCanCope with PostOperative Pain (“iCanCope POP”) is a smartphone app that provides adolescents with evidence-based strategies to manage postoperative pain. The goal of this study was to explore the unique needs and pain-management experiences of youth undergoing cardiac surgery to inform the development of a tailored iteration of iCanCope POP.

**Methods:**

A descriptive qualitative design using semi-structured interviews and focus groups was conducted with adolescents ages 12–18 who had received cardiac surgery, caregivers of youth undergoing cardiac surgery, and interdisciplinary healthcare providers (HCPs). Qualitative data were inductively and deductively coded using a content analysis approach to outline participants’ pain management experiences and perspectives on the iCanCope POP app.

**Results:**

14 interviews and 1 focus group were completed with 6 children, 6 parents, and 12 HCPs (*n* = 24 participants). Content analysis resulted in 3 key categories: 1. Riding the rollercoaster of the surgical journey (e.g., ups and downs of pre- and post-operative phases); 2. Postoperative pain experience (e.g., tubes, technologies, and devices; pain management strategies); and 3. App feedback (e.g., usefulness, appropriateness, suggested modifications).

**Discussion/conclusion:**

Participants reported significant challenges during the perioperative pediatric cardiac surgery journey and identified potential opportunities for a tailored mobile self-management app to improve their surgical experience. Future research should use these perspectives to inform a new iteration of the iCanCope POP app for youth undergoing cardiac surgery.

## Introduction

Congenital heart disease (CHD) is the most common birth defect globally, accounting for nearly one-third of all congenital defects and affecting approximately 1% of live births [1,2]. Youth with complex CHD require ongoing treatment, procedures, and surgeries over the course of their lifetime to maintain optimal cardiac functioning [3]. These cardiovascular surgeries can cause both physical and psychosocial sequelae; youth can also experience challenges related to mental health which can interfere with social functioning [4–7]. Compared to adults [8], a higher proportion of children (>20%) who undergo cardiovascular surgery (CVS) report persistent pain from 3 months to four years postoperatively [9,10]. Adolescents who experience chronic pain also tend to have high self-reported levels of disability, depression, and anxiety [11], which can persist into adulthood causing long term disability [12]. There is also evidence that brain responses evoked by pain can be greater in adolescents compared to adults, in the brain regions important for affective and cognitive processing [13]. Developing resiliency and coping strategies in youth is critical for reducing the burden of chronic pain in adulthood [14], so this age group is a key target for postsurgical pain prevention.

iCanCope with Pain is an evidence-based pain self-management app for youth that was developed by an interdisciplinary team of pediatric pain experts and was originally designed to support youth living with chronic pain [15]. The iCanCope app delivers a holistic self-management program informed by established behavior change theories [15–17] through three core components: (1) Symptom tracking – a daily “check-in” where users can monitor pain intensity and related symptoms such as activity limitations, sleep quality, mood, energy, and movement; (2) Personalized goal setting – tools to set SMART goals (Specific, Measurable, Achievable, Realistic, Timely) aimed at improving pain levels or functional abilities; (3) A library of pain self-management strategies (e.g., relaxation exercises, deep breathing, mindfulness, cognitive reframing) [15–17]. The platform has since expanded to be tailored for specific painful conditions such as arthritis [16], sickle cell disease [17], and neurofibromatosis [18] and was further adapted to meet the unique challenges faced by youth recovering from surgery, creating the iCanCope PostOperative Pain (POP) app [19]. The iCanCope POP app supports the adolescent postoperative population by presenting evidence-based strategies in an accessible and engaging format to help users better understand their condition and track symptoms such as pain, sleep, mood, and activity, and visualize symptom trends. Due to the highly specialized nature of pediatric CVS, patients may have limited access to specialized postoperative support in the community, indicating the value of tailored virtual support programs in this population.

Digital health interventions have been shown to improve quality of life by reducing symptom burden, improve medication adherence, reduce healthcare utilization, enhance symptom monitoring, and increase patient satisfaction for adults undergoing CVS [20]. However, there are no existing evidence-based digital health interventions for improving pain and symptom management for youth undergoing CVS. To address this problem, this project sought to understand the unique needs of youth and families undergoing CVS and describe how an existing smartphone-based self-management application (“iCanCope PostOperative Pain”) could be modified to better support this population.

## Objective

The goal of this study was to qualitatively explore the unique needs and perspectives of youth aged 12–18 undergoing cardiac surgery, their caregivers, and their healthcare providers (HCP) to understand how the current iCanCope POP app could be modified to better support young people undergoing CVS; this is the first phase of a multi-phase study.

## Methods

### Study design and setting

This study employed a generic qualitative descriptive design [21,22] with a content-analysis approach, using semi-structured, audio-recorded interviews, and focus groups with children, parents/caregivers, as well as HCPs. Participants were recruited from the Hospital for Sick Children CVS Department. This study received research ethics board approval (#1000080243).

### Inclusion and exclusion criteria

A purposive sample of patients and caregivers was recruited, aiming for diversity in age, gender, ethnicity, and other socio-demographic factors. Adolescents were eligible if they (1) were 12–18 years, (2) had undergone cardiac surgery with median sternotomy in the past 3 months, (3) experienced acute postoperative pain of any intensity according to self-report, and (4) were able to speak and read English. They were excluded if they (1) had undergone transplant surgery, (2) had severe cognitive impairments, (3) had major co-morbid medical or psychiatric illnesses (e.g., severe anxiety disorder) that could preclude their ability to participate in a verbal interview, or (4) had an existing chronic or recurrent pain condition. The sample age of 12–18 years old was selected because adolescents are in a critical developmental period where rapid changes take place in the brain, body, and behaviors [23]. Adolescents who experience chronic pain also tend to have high self-reported levels of disability, depression, and anxiety [11], which often persist into adulthood causing long term disability [12]. It has been estimated that 20% of adolescents develop chronic postsurgical pain [24], highlighting a critical need to address pain particularly in this population in order to prevent long-term impacts.

Parents were eligible if (1) their child met all the inclusion criteria and none of the exclusion criteria, and (2) they were able to speak and read English. They were excluded if they had (1) severe cognitive impairments or (2) major medical or psychiatric illnesses that could preclude their ability to participate in a verbal interview.

HCPs involved in the management of patients undergoing CVS were recruited to participate in audio-recorded semi-structured focus groups or individual interviews. We aimed to include a sample that varied across profession and level of experience; HCP were eligible if they (1) had worked within pediatric CVS for at least 1 year, and (2) could speak and read English.

### Data collection

All eligible patients (*n* = 42) were introduced to the study through an information letter at their preoperative appointment and then were followed up via their care team postoperatively. Clinical team members who were supporting recruitment had ongoing meetings with the study team to support a purposive approach to sampling to review sample demographics. Clinicians were briefed on the aim to include diverse participants who varied across characteristics such as age, ethnicity, socioeconomic status, medical and surgical history, etc. Youth and parent participants were eligible to participate as a dyad or individually. Seven youth participants received the consent form; one dropped out prior to consent and six youth participated, along with six parent/caregiver participants. Data was generated and analyzed iteratively, and after interviewing 12 youth/caregiver participants, the research team shifted recruitment efforts to target HCPs to further triangulate findings. HCPs were recruited through a study information letter. After conducting an in-person focus group with 8 HCP participants, it was determined that some HCP perspectives were still missing so 4 additional HCP participants were purposively targeted to participate in one-on-one interviews. At this point (*n* = 24), data saturation was established, with several recurrent findings across all three participant groups.

Consent and data collection occurred remotely or in person; participants completing a demographic questionnaire via REDCap and then participated in individual interviews conducted by a trained qualitative interviewer (LH), who has experience conducting qualitative digital health research and was not directly involved in the patients’ care. Interviews were conducted using Microsoft Teams. The interview guide was pilot-tested with youth partner (MS). Interviews were scheduled for 1 hour, and the average length was 47 mins (range 35–53 minutes). During interviews, participants were either given access to the iCanCope POP app or provided with a virtual demonstration of the app (see Appendix A – Screenshots of iCanCope POP app). HCP focus groups were facilitated by two trained research team members: one facilitator moderated the focus group discussion, while the other took detailed field notes. The clinician focus group length was 68 minutes. If a HCP could not attend the focus group, an individual interview was arranged (*n* = 4).

### Data analysis

Demographic and disease-related data were analyzed using descriptive statistics with logic and range checks employed to verify the accuracy of the data. Interview audio-recordings were transcribed verbatim and Dedoose software [25] was used for data management and coding. Throughout data generation and early analysis, two team members (LH, TK) read full transcripts to become familiar with the data and met weekly to reflexively conceptualize patterns and develop key concepts for the codebook. A combination of inductive and deductive content analysis was used; [21] codes were developed deductively based on concepts from previous research on the iCanCope platform and interview guide, as well as inductively using the participants’ own language. After the initial codebook was established, two team members (LH, ND) coded two transcripts from each participant group (youth, parent, HCP). Codes were compared and modified, and the codebook was restructured to account for new data as coding progressed. Categories and sub-categories were then generated by grouping the codes and developing an account of the surgical and pain-management experiences across all three perspectives (youth, parent, HCP). A team of coauthors (TK, LH, ND, JS) reviewed the categories and sub-categories and contributed to the development of descriptions. Categories were finalized by writing up results and weaving participant data (quotations) along with interpretations; this process allowed for an understanding of pain self-management needs and optimal design suggestions informed by the experiences of participants. Quotations from a range of participants were included to ensure a variety of perspectives were represented across each category.

#### Reflexivity

Data generation and analysis was conducted by researchers who hold a range of backgrounds (i.e., nursing, cardiology, CVS, anesthesia, research, lived experience of surgery and pain, etc.). Reflexivity was incorporated throughout the research process both individually and as a group; the team routinely discussed their relationship to and interest in the research topic, and examined how their professional and personal positionality influenced the interpretation of data throughout the analytic process. Specifically, the first author is a PhD-prepared cardiac nurse with clinical expertise in adult cardiac surgery and cardiology, which brought a unique perspective to the exploration of youth and family dynamics within the cardiac surgery journey.

## Results

Demographic characteristics of adolescents (*n* = 6), parents (*n* = 6), and HCP (*n* = 12) are summarized in Table 1.

**Table 1.** Sample demographics

Characteristics	Adolescents (*n* = 6)	Parents (*n* = 6)	Health care providers (*n* = 12)
**Sex, *n* (%)**			
Female	3 (50)	5 (83)	10
Male	3 (50)	1 (16)	2
**Age (years), mean**	14.33	–	–
12, *n* (%)	1 (16)	–	–
13, *n* (%)	0 (0)	–	–
14, *n* (%)	2 (33)	–	–
15, *n* (%)	2 (33)	–	–
16, *n* (%)	1 (16)	–	–
**Ethnicity, *n* (%)**			
Indigenous (Inuit, Metis, North American Indian)	1 (16)	–	–
Black (e.g., African, Haitian, Jamaican, Somali)	1 (16)	1 (16)	–
White (Caucasian)	4 (66)	4 (66)	–
Other (Jewish)		1 (16)	–
**Type of cardiac surgery, *n* (%)**			
Aortic root replacement (bentall)	1 (16)	–	–
Conduit RV-PA replacement	1 (16)	–	–
Mitral valve replacement	1 (16)	–	–
Ross procedure	2 (33)	–	–
Subaortic stenosis repair	1 (16)	–	–
**Length of hospital stay postoperatively, *n* (%)**			
0–3 days	2 (33)	–	–
4–6 days	4 (66)	–	–
**Type of expert, *n* (%)**			
Child life specialist	–	–	2 (17)
Nurse practitioner/advanced practice nurse	–	–	1 (8)
Occupational therapist	–	–	2 (17)
Physical therapist	–	–	2 (17)
Physician	–	–	3 (25)
Registered nurse	–	–	2 (17)
**Years of experience as a health care provider, *n* (%)**			
0–5	–	–	2 (17)
6–10	–	–	2 (17)
11–15	–	–	2 (17)
16–20	–	–	2 (17)
20+	–	–	4 (33)

The results are organized into three major categories which outline various aspects of the postoperative process for pediatric cardiac surgery patients, particularly as it relates to pain management and potential app utilization: (1) Riding the rollercoaster of the surgical journey, which was stressful for both youth and family members as they navigated challenges and unanticipated setbacks; (2) Postoperative pain experience, which focused on the devices and therapies that both produced and help to relieve pain after cardiac surgery; and (3) Overall app feedback, which highlighted the unique needs of adolescents across the perioperative journey of cardiac surgery. A supplementary file of representative quotes for each category can be found in Appendix B – Supplementary Quotes.

## Category 1: Riding the rollercoaster of the surgical journey

Participants described the surgical journey as a stressful and anxiety-inducing experience, with challenges and unanticipated setbacks. The preoperative phase focused on preparation and expectations for surgery, centering around the key milestones of the preoperative appointment and subsequent surgery, whereas the postoperative phase focused on challenges of the hospital stay, variations in recovery, and the return to daily life.

### Preoperative experience

The preoperative appointment, which typically occurs 4–8 weeks prior to surgery serves as a critical touchpoint for information exchange between providers and families. However, our analysis suggests that the volume and timing of information can feel “overwhelming” for youth and families, which can inhibit their ability to absorb crucial information. Participants also reported forgetting information due to the length of time that elapsed between the preoperative visit and the surgery itself:

What happened with me, my preop was two months before my actual surgery,[…] I forgot a lot of it or didn’t really think of it. (14 year old male patient)

Feelings and expectations about surgery varied widely, and often discussed preoperative concerns related to pain, recovery, and potential complications:

He was more concerned about the scar and the pain after, you know, he was more concerned about that, that was the only two things that really bothered him. (Parent)

Worries galore and you know, just worries, of course, of, you know, something going wrong. (Parent of 16-year-old male patient)

Unplanned delays and prolonged stays further exacerbated these challenges, especially postponed and rescheduled procedures.

My surgery was postponed like…a week and then a day after we went to the hospital and the day of my surgery, it got postponed like a day after. […] I never got any information about how I should cope with my anxiety during the time. (14-year-old female patient)

In response to anxiety, several participants shared coping mechanisms for managing stress and discussed a range of self-directed distraction strategies:

Take your mind off it, like going for a short walk or…[d]rawing, reading anything that could take your mind off it in that form like media, because you will just get more anxious. (14-year-old female patient)

I tried to get outside pretty frequently, like I got outside for four or five hours a day. …Pretty much I was golfing like every day for the week before. (14-year-old male patient)

Participants experiences highlighted the emotional weight carried by families as they anticipate surgery and the factors affecting this preoccupation. Overall, our analysis suggests that the preoperative experience can be a stressful period for youth and their families, and HCP play a vital role in guiding them through this phase, by providing information, fostering collaboration and addressing emotional concerns.

### Postoperative experience

The postoperative experience is marked by physical recovery, emotional adjustment, and the navigation of various healthcare supports. Following surgery, youth and families were focused on following a routinized recovery process where they moved from intensive care unit to surgical ward and then discharge, typically within 3–5 days. Our analysis yielded themes of realistic goal setting and the collaborative nature of care, where providers and families work together to achieve positive outcomes:

Our expectation is they’re up, they’re about, they’re walking. They’re not just lying in bed, then that’s how we get them out of the hospital that quickly. (Physician)

Many participants revealed that recovery was often nonlinear, with unexpected setbacks adding to the emotional and physical burden. Youth and caregivers shared feelings of unpreparedness regarding recovery and challenges understanding the magnitude of surgery:

It hadn’t really been talked about that it probably will be an up and down process as opposed to, like, there was a lot to talk about pain management and stuff, but he wasn’t expecting to kind of have the bumps in the recovery and that would have been helpful for him to have had that awareness, that preconceived notion going into it, because I think again he would have been, you know, better mentally prepared for it. (Parent of 14-year-old male patient)

So when everything was happening, I think I kind of underestimated how, uh, big the process was so, uh, I think I could have cared a little bit more about how big this was, yeah. (15-year-old male patient)

Complications often led to delayed discharge or even a return to critical care units and participants described having to adjust their postoperative course or care plan. Families were also surprised at the rapid discharge process:

…at the end it was as much as we wanted to leave […] there was a real feeling of “are you sure we should be leaving right now?” It just seemed very weird. I’ve been telling friends, you know, one day, someone’s holding your heart in their hand and fixing it. And two days later, you’re walking out of the hospital like it just seemed very, very weird to a non-medical person who doesn’t see it every day. You know, it’s just shocking that that’s yeah. So it feels like you should be in the hospital for a month or something. (Parent of 16-year-old male patient)

Once patients were ready to be discharged home, the transition from hospital to home was often accompanied by anxiety and uncertainty. Caregivers conveyed mixed feelings about this transition, particularly regarding access to clinical support as many families do not live near a specialized care center, adding to the burden of recovery and medical decision making:

It was also nerve-wracking because going home with such a major surgery can be and we don’t live close to a hospital. We’re quite rural, so our nearest hospitals aren’t really equipped for children, let alone, heart…our nearest hospital is Hamilton, which isn’t too bad. It’s about a 45-minute drive. (Parent of 12-year-old female patient)

Finally, participants described their return to daily activities, such as sports and school, as a significant milestone in the recovery process, accompanied by a mix of relief and adjustment. Overall, our analysis demonstrated the multifaceted nature of the surgical journey that extends beyond physical recovery to encompass emotional and logistical dimensions. Unexpected setbacks can further complicate recovery, and participants sought more information about the variations in postoperative courses and how to cope with the impact of CVS on the family unit.

## Category 2: Postoperative pain experience

Our second category focuses specifically on the pain experience following pediatric CVS and explores influential factors such as family dynamics, self-care activities, and pharmacological approaches to pain management.

### “Tubes and technologies:” impact of devices on pain experience

Following CVS, participants described the acute postsurgical pain as primarily concentrated in the first few days following surgery, with a gradual decrease as the days progressed:

I was only there for a few days and it was kind of painful for like those first, like three or four days. (16-year-old male patient)

Chest tube placement was a significant source of pain for many patients, and it was recognized that chest tube removal was a key milestone in recovery:

I think that the most pain they have is when the chest tubes are in, so getting the chest tubes out are really important and the feedback is, is that when the chest tubes come out, they are, it is much better. (Nurse Practitioner/Advanced Practice Nurse)

Moreover, several HCP noted that increased levels of reported pain in the immediate postoperative period are typically associated with the duration of chest tube and therapeutic device placement. Management of chest tubes also impacted the pain management approaches by the HCP team:

There have been times where sometimes some of these valve replacements we do have to keep their chest tubes in longer. They don’t want them out like on post-op day one and they want them in a little bit longer. And sometimes I cannot get on top of their pain, and sometimes I do have to call the pain team just to help, like, get a plan. (Nurse Practitioner/Advanced Practice Nurse)

HCP also noted that in their experience, surgeries with a thoracotomy approach were more painful than a sternotomy approach, however the primary cause of pain was still related to the presence of chest tubes or other invasive therapies:

I think the surgeries probably do matter, but I think maybe less so about the surgeries themselves. But I think what’s left behind after the surgery. So how long the chest tubes are in what kind of tubes and the technologies are left to look behind in the body. (Physician)

Overall, participants described the pain journey as being focused on the immediate postoperative period and centered around the presence of devices.

### Pharmacological, physical strategies, and psychosocial strategies for pain management

Several pain management strategies, experiences, and routines were described by patients and HCPs that were used to manage pain both in hospital and at home in the postoperative period. Regarding pharmacological management, HCPs emphasized the importance of maintaining routine pain medication administration especially in the acute postoperative phase:

They all get IV pain meds in the ICU. And then when they come upstairs, we transition it to oral. But we keep them on meds regularly so that they’re not having gaps in coverage because obviously as they get better, they’re going to mobilize more (Physician)

Moreover, patient participants discussed using various techniques such as splinting (“coughing to use a pillow like squeezes around my chest so the pain doesn’t hurt as much”) and distraction (“I just watched a bunch of basketball to keep everything that was going on in the hospital off my mind) as alternate methods of pain management. Using a biopsychosocial approach to manage postoperative pain encouraged patients to engage in self-management during recovery.

### Family dynamics and perspectives on surgery and pain

Family views on medication use for pain relief greatly influenced patients’ care trajectory and pain experience both in the hospital setting and at home. In some cases, patients and families may limit pharmacological solutions, resulting in ineffective pain management:

I think we kinda go two ways at home too with the medication. I think there’s some patients and families that take too little. You know, especially in those early days at home and we know it’s certainly in our experience much harder to catch up if you do that (Physician)

Moreover, HCP described situations where parents may not “believe in pain” leading to inadequate pain management, in addition to a fear of the child developing an opioid dependency or addiction which could act as a factor limiting appropriate analgesic administration:

Other factors are always those going to be the parents that just don’t believe in pain, the pain experience then don’t advocate for their child based on what we tell them. Like there’s the fear of like addiction. (Nurse Practitioner/Advanced Practice Nurse)

In response to this, HCP play a crucial role in managing client and family expectations preoperatively:

I’m trying to again be pretty frank that like they are going to be in pain afterwards, but like, we rely on like you guys as parents to help encourage what we are trying to encourage at the bedside. Because without your buy-in, like things really don’t progress as well as we would like them to. (Physical Therapist)

In addition to the role of parents, one participant noticed that it was their child who was reluctant to report their pain due to fear of lengthened hospitalization:

I think she thought if she was in pain she wouldn’t be able to go home as fast. So she covered it up and said she wasn’t. (Parent of 12-year-old patient)

Ultimately, within the pediatric population, the impact of both adolescent and parent beliefs played a significant role in pain management strategies and the overall pain experience.

### Self-care activities (i.e., sleep, nutrition)

The postoperative period was a challenging time for some participants; they struggled with engaging in self-care activities such as sleeping and eating, highlighting the emotional and physical impact of the hospital environment on the pain experience:

When I was in the hospital, I didn’t really sleep much, but when I got home and I was like in my own bed, I kind of just I found it really easy to sleep. (16-year-old male patient)

Because he was in the hospital for so long, it wasn’t just the incision that was hurting. Then his back started to hurt a lot and he had a lot of trouble sleeping sometimes because of the back pain. (Parent of 15-year-old male patient)

In terms of nutrition, families noted concerns regarding how their nutrition would change post-procedure which provides an opportunity for additional education:

The nurse would just like pop in and say like, eat your food or like try to eat or something. But like it was kind of like hard to eat. (16-year-old male patient)

HCP highlighted that those who receive adequate nutrition and have “good sleep hygiene” prior to surgery may have better recovery experiences. Overall this emphasizes the role of presurgical preparation and educating youth in self-management strategies in advance of the surgery to support their postoperative recovery.

## Category 3: App feedback

This final category was shaped by a discussion of resources that could be supplemented by the mobile iCanCope POP app as participants reflected on the existing features of the app and potential modifications or areas of improvement to tailor to the CVS population. A summary of proposed changes to the app that would allow the platform to be tailored to the unique CVS population is outlined in Appendix C: Summary of Proposed App Modifications.

### Goals, check-ins, and trends

Participants appreciated the goal setting feature embedded in the app and suggested that reminders for certain aspects of recovery such as medication administration or exercise reminders be included. Participants requested the development of user options to determine frequency and type of reminders to improve individual engagement with the app:

That’d be super helpful at home for kids, I think, because you know, like [Patient Name], is old enough and smart enough for me to be able to say, OK, you can take this again in 4 hours. And this again in six hours, you know. (Parent of 14-year-old male patient)

Moreover, positive feedback was received from several participants regarding the check-in feature. The different categories of assessment were noted to be useful and participants appreciated the opportunity to provide numerical assessment scale. This feedback extended into the displaying of data trends within the app in relation to pain, mood, and others. Several participants noted that displaying trends could act both as a motivator and an assessment tool for parents or the clinical team regarding pain and other psychosocial elements:

It would show that you’re making progress, which is nice because at times I wasn’t really sure that I was really making that much progress. And it was hard to compare what I was doing to previous days and that first stuff. So having a graph there that visually showed it would definitely help. (14-year-old male patient)

I think mood would be the most important just because even she, like she wouldn’t talk to us about how she was feeling but I feel like maybe she would put it on there, how she’s feeling. (Parent of 12-year-old female patient)

Ultimately, participants reported that iCC POP app features such as monitoring trends, daily check-ins, and goal setting would be helpful during the recovery process for CVS and provides an intuitive approach to monitoring and supporting postoperative recovery.

### Resource library

In addition to providing feedback on existing app features, there was emphasis placed on editing the library of resources to better address the needs of this patient population. Participants discussed tailoring information based on high-touch (i.e., immediate postoperative) and low-touch (i.e., post-acute phase) periods and providing an overview of what participants can expect and work towards during these phases:

I think like the resource perspective is going to be the most important thing to say, like, […] you are now post-op day two, like you might still be experiencing some throat pain from intubation, extubation, you might still have some incisional pain, with reminders that it’s important to continue to do deep breaths. Like whatever. That stuff will be nice to kind of map out for them that like, again, it’s OK for you to still be uncomfortable. (Physical Therapist)

It’s nice to be able to integrate the, you know, the bathing and incision care piece into there as well. […] (Physician)

Moreover, patients and parents alike identified the potential for reemphasizing surgical recovery expectations in all spheres (psychosocial, physical, etc.) through the app so it may be leveraged as a tool during recovery (i.e., to support sleep hygiene). Overall, this feedback demonstrated the need to tailor educational resources to support user engagement and improve both the preoperative preparation and the postoperative recovery experience.

### Potential features and overall impressions

Lastly, participants shared their overall impressions on the existing iCC POP app in relation to their experience with CVS to determine if any additional features could be added to better support this patient population during their recovery.

One of the potential features suggested was guided breathing exercises (i.e., a video demonstration of incentive spirometry with accompanying timer):

That’s important to have in the app as well as maybe an outline of some of the expectations after surgery, like there was a few times where we were told by one nurse that somebody would come in to see us, but then they didn’t. And it was about like breathing exercises. So that’s something that we didn’t necessarily need somebody to take their time to come show us if it was on an app. (Parent of 12-year-old female patient)

I was kind of just like taking it day by day. But I just, well, my goal I guess was like just to do my exercises every day and like and then like my breathing exercises too. Like just try to, uh, keep on doing that every day and I’d say it worked pretty well. (16-year-old male patient)

Another potential feature suggested was the inclusion of perioperative checklists. HCP suggested that the app’s ideal introduction would be during the preoperative period, which would help to ensure users have access to resources prior to their procedure. Several components were highlighted as potential additions such as preoperative checklists, plans for post-op preparation, and other educational resources:

I guess to me it would make sense to be trying to at least introduce this in the pre op visit […] maybe it’s even possible that it gets written into the information that goes out to them, so that for those interested patients and families, they can even look around at it even before they come to pre op and, and have that reinforced […] And then and then having that and then being able to go back over it again in that in that the brief stay that many of them have post operatively. (Physician)

Parents and patients expressed the addition of a question-and-answer section in the app (i.e., FAQ or chatbot) could be a powerful tool to support families during both pre- and post-operative periods. This feature could primarily serve to minimize barriers families may have with regards to accessing the clinical team.

Like if you have any questions like maybe a little chat area, like if they if you have any questions you can add in, somebody can get back to you or you know you can add a little picture or something just and get an info quickly back or an answer quickly back that would be great. (Parent of 15-year-old female patient)

Another unique finding is that participants saw the introduction of a focused version of the iCC app as an opportunity to empower young patients to be in charge of their own health and develop independence and self-management skills:

And a point I just wanted to make is that I see another benefit of using the application. There are a lot of teens who are not very engaged in their own health care, mostly like this is done by the parents. So, this way they can become more engaged. And more knowledgeable. And this would be helpful in the process of transitioning to adult care as well, (Nurse)

Finally, participants did comment on some potential challenges or minor concerns related to using an app for support. One parent wondered if youth might access information in the app that doesn’t apply to them (i.e., information about a different surgery, etc.). Another parent expressed doubt that their child would access the information in the app, but saw value in parental access:

We [the parents] can go through the app and we can and look for stuff and say hey, the articles in here or because I know he’s a teenage boy, he’s not gonna read it, but even if I could read it and get the points for him and say, hey, this is the answer to your question, you know what I mean? (Parent participant)

In the focus group, clinicians noted that patients can often feel overwhelmed with information in the immediate postoperative period, especially when youth are discharged rapidly, and suggested that information be introduced in the preoperative period, which aligned with findings from the youth and parents perspectives.

Altogether, this feedback lays the blueprint for adjusting the iCC POP app to better serve pediatric patients undergoing cardiac procedures and improve their engagement in their own healthcare going forward.

## Discussion

Undergoing major CVS can trigger significant stress for adolescents and family members, as reflected by participants in this study. Due to the complexity of pediatric CVS, participants require comprehensive support both pre- and post-operatively to manage pain effectively and promote optimal recovery. A recent position statement by the American Heart Association noted that adolescents and their parents require detailed information about the upcoming procedure, expected outcomes, and potential complications to reduce anxiety, improve cooperation, and enhance post-procedure recovery [26]. Additionally, the authors report that teaching coping skills prior to surgery has proven effective in improving in-hospital and post-discharge adjustment [26]. This coupled with our findings suggests that additional self-management strategies and education in the form of a mobile app may be a beneficial approach to support youth and their families. A mobile pain-management app can deliver multifaceted support, offering preoperative education, coping skill training, and real-time guidance on pain monitoring and medication adherence. Given that pain can persist for weeks after surgery, ongoing support is necessary [27], and an app can provide an accessible solution to bridge the gap between the hospital and home.

Acute postoperative pain, including neuropathic pain, is prevalent in pediatric patients undergoing various major surgeries including cardiac, orthopedic, and abdominal [28,29]. However, there are unique considerations for cardiac surgery related to pain management. Pediatric cardiac surgery involves sternotomy or thoracotomy incisions which are associated with muscular pain that can interfere with deep breathing and coughing; the placement and ongoing presence of chest tubes can also be a source of discomfort. Another unique perspective that emerged from this population compared to patients receiving orthopedic surgeries were concerns related to body image and specifically how the scar would look on their chest. In addition to pain, youth undergoing a variety of surgeries report concerns regarding with mental health (e.g., anxiety) as well as sleep disruption which can impact their postoperative recovery; [24,30,31] this aligns with the findings of this study and the concerns of patients receiving cardiac surgery. The majority of features available on the iCanCope POP app remain relevant for cardiac surgery, however additional suggestions emerged (i.e., the need for guided incentive spirometry demonstrations, the need for an overall surgical “pathway” to guide patients through the perioperative journey) which will be developed in the modified app for cardiac surgery.

Through this study we identified that a digital health app could be beneficial in the pediatric CVS population and have highlighted several modifications or additional features that could enhance perioperative support for this unique group (summarized in Appendix C). Specifically, participants would like tailored pre- and post-operative CVS information and education regarding preparation and recovering from surgery, as well supported rehabilitation interventions such as video-guided incentive spirometry and medication administration reminders.

Participants reported that a mobile self-management app could make a valuable addition to their perioperative journey, which aligns with results from a recent review of digital health tools for adults undergoing CVS showing that digital health interventions were associated with improved patient engagement, satisfaction, and reduced healthcare utilization [20]. Results of this review indicated that patients felt digital interventions were helpful in their recovery, and showed evidence of enhanced symptom monitoring and timely intervention [20]. Patients also expressed that these interventions resulted in improvements in mental health, quality of life, and eHealth literacy [20]. Another recent study evaluated the feasibility of using a mobile health app to deliver Enhanced Recovery Protocol information to cardiac surgery patients [32]. The app achieved a 75% utilization rate, with high patient satisfaction, demonstrating the feasibility of use and high rate of uptake of digital interventions in the adult CVS population [32].

While these advancements demonstrate the efficacy of digital health solutions for adults who have undergone cardiac surgery, there remains a notable lack of evidence-based self-management apps for improving pain and symptom management for youth undergoing cardiac surgery. This gap underscores the importance of developing mobile pain-management tools specifically designed for younger patients, as their needs, engagement preferences, and recovery experiences differ significantly from those of adults. Our study fills an important need in the field by taking the first step towards developing and testing a comprehensive smartphone app to assist in the management of acute postoperative pain among children and adolescents undergoing cardiac surgeries. Patients benefit from this iterative approach as the iCanCope app was originally developed through user-centered design, and has been effectively tailored to address the unique needs of patients in other disease groups (i.e., sickle cell disease, juvenile arthritis, etc.). Considering the needs of youth undergoing CVS, this iterative approach allows for modification and testing of an effective solution which will support patients sooner than having to develop an original app. This approach also benefits from existing validated back-end infrastructure, design, functionalities, and sustainability of the larger iCanCope platform which will enhance uptake and long-term maintenance. Overall, the current literature aligns with the findings from this study that patients are interested in using digital tools that support perioperative self-management to provide meaningful improvements the surgical journey.

## Limitations and strengths

All participants were recruited from a single site (SickKids), the leading cardiac surgery center in Ontario and the sample was limited to only those undergoing CVS via sternotomy; therefore, the sample was limited to those patients who fit the remaining eligibility criteria within the timeframe of the study. In addition to this, despite recruitment efforts, there was minimal diversity in socio-demographic characteristics of participants in the sample, which may limit transferability to other settings. Specifically, the risk of including a homogeneous sample is that the development of interventions that will be tailored to this population and may not be as effective for youth who do not fit these characteristics. It is therefore critical that diverse partners with lived experience remain involved in the design and iterative development phases, as well as the evaluation phase. Future design and evaluation phases will include recruitment strategies specifically designed to engage underrepresented groups, to ensure relevance and acceptability across diverse audiences. In the context of this study, including parents and HCP as well as youth in the sample allowed for a variety of perspectives to be triangulated and synthesized on the critical perioperative cardiac surgery experience.

## Conclusion and future directions

In conclusion, youth, parents, and HCPs reported significant challenges during the perioperative pediatric cardiac surgery journey, ranging from preoperative stress and anxiety to postoperative pain management and recovery. Participants identified the potential benefits that a tailored mobile self-management focused app could have on their surgical journey and outlined specific opportunities to modify the app to meet the unique needs of this population. Future directions will focus on co-designing app features, conducting usability testing with a diverse sample of participants and evaluating the clinical effectiveness in managing postoperative pain for youth undergoing cardiac surgery. Ultimately, we aim to prepare the app for public deployment, where it can be widely adopted as a reliable, evidence-based tool for postoperative CVS pain management.

## Supporting information

10.1017/cts.2026.10741.sm001 Killackey et al. supplementary material Killackey et al. supplementary material

## Supplementary material

The supplementary material for this article can be found at https://doi.org/10.1017/cts.2026.10741.
