# Supplementary material for: Exploring the perioperative experiences of youth undergoing cardiac surgery: A qualitative needs assessment for tailoring a mobile self-management app
Source: J Clin Transl Sci. 2026 Apr 22;10(1):e85. doi: 10.1017/cts.2026.10741 (PMC13202492; doi:10.1017/cts.2026.10741)
Supplement: Killackey et al. supplementary material [file S2059866126107419sup001.docx]

**Supplementary Materials**

**Appendix A: Screenshots of iCanCope POP app**


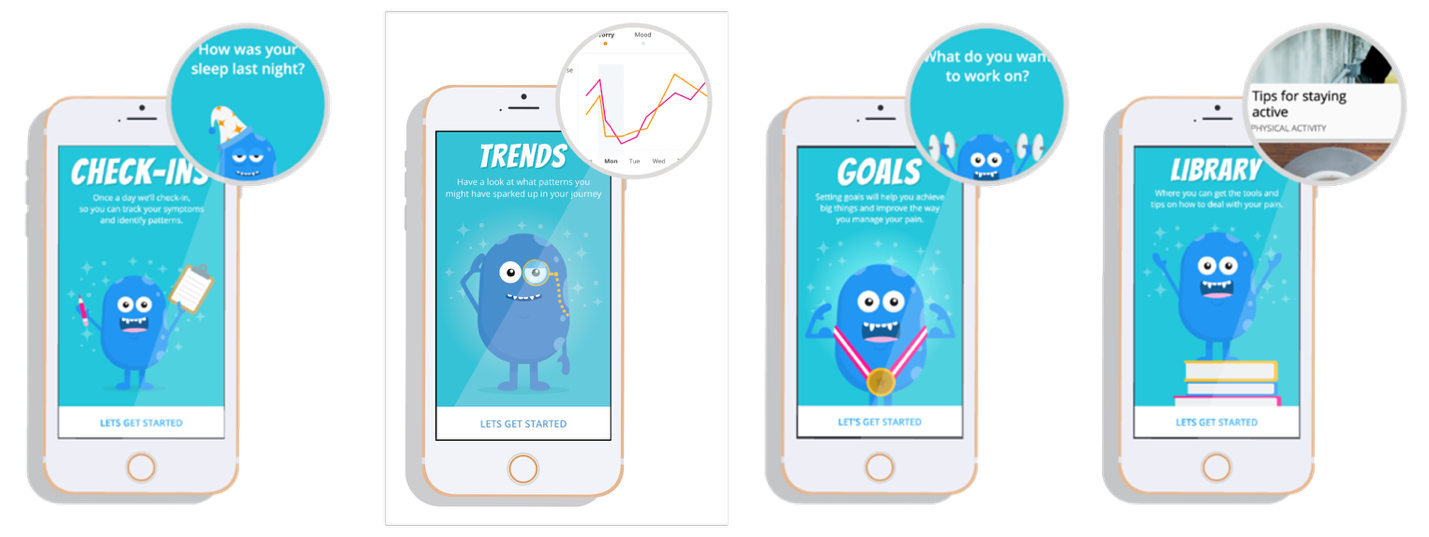


**Appendix B: Additional Representative Quotes**

| **Category** | **Sub-Category** | **Quote** |
| --- | --- | --- |
| **Category 1: Riding the rollercoaster of the surgical journey** | ***1.1 Pre-operative experience*** | “it kind of felt overwhelming. So my brain just kind of blurred out most of it.” (14 year old female patient) |
|  |  | “having the nine different people come and talk with us was fantastic. You know, there really was no opportunity there for us not to ask questions.” (Parent of 16 year old male patient) |
|  |  | “I never got any information about how the how I should cope with my anxiety during the time.” (14 year old female patient) |
|  | ***1.2 Post-operative experience*** | “she loves her sports and whatnot. And then she just noticed that she was getting tired more often out of breath, more often. So after the surgery, that was kind of our focus like being happy that she can somewhat be normal again and get back into activity without getting so tired.” (Parent of 15 year old female patient) |
|  |  | “I don't know, there was a pocket of fluid, I think, or something that. And he was just really uncomfortable. And then there was some, you know, extra drainage that was happening or whatever, like it just. And he was in a lot of pain and we had been moved to the floor already…And they actually moved us back down to the CCU just to be cautious, and because his level of attention at that time was too much for the floor.” (Parent of 14 year old male patient) |
|  |  | “He has a mechanical valve, so his INR had to get to a certain point for him to be discharged. So in order for him to do that, he had to eat and keep down food at a certain amount of food…the goals while he was there was like he had to eat, say, breakfast, or he had to eat lunch or he had to eat dinner and keep a certain amount down because there was medication that they need to give him and they weren't able to give him orally until because he was just throw it up.” (Parent of 15 year old male patient) |
|  |  | “we don't know what our judgment call is. And I guess that's why going to the hospital the week after or [...], you know, having your doctor's appointment two weeks after whatever it was beneficial.” (Parent of 16 year old male patient) |
|  |  | “they went over the information with me page by page and asked me if I had any questions. And so that was that was very helpful…I think they were very thorough before we left. So myself and my husband, I think we felt very comfortable.” (Parent of 15 year old male patient) |
|  |  | “I think that was one of the main ones was just getting independent, you know, being able to get up on my own and transfer on my own and even walking because he wasn't allowed to walk on his own either without the walker, so he graduated to his canes very quickly and all his working on he wants to get rid of the canes.” (Parent of 15 year old male patient) |
| **Category 2: Post-operative Pain experience** | ***2.1 "Tubes and Technologies”: Impact of devices on pain experience*** | The one thing that we've noticed is that the thoracotomies seem to be more painful than the sternotomies.[…] I'd say a pacemaker's less painful than a sternotomy or thoracotomy. I think in general that's what we see” (Physician). |
|  |  | “Typically getting out the chest tube, or tubes that get put in, all of our surgeries makes just a huge difference to their pain, and so we're trying to really focus on doing that really, really early as often as we can” (Physician). |
|  |  | Yeah, like so, he had chest tubes in and and like, there was like, I don't know, there was a pocket of fluid, I think, or something that. And he was just really uncomfortable. And then there was some, you know, extra drainage that was happening or whatever, like it just. And he was in a lot of pain” (Parent of 14 year old male patient) |
|  |  | The one thing that we've noticed is that the thoracotomies seem to be more painful than the sternotomies. That's one thing that we've noticed.[…] I'd say a pacemaker's less painful than a sternotomy or thoracotomy. I think in general that's what we see” (Physician) |
|  | ***2.2 Pharmacological, Physical Strategies, and Psychosocial Strategies for pain management*** | “The pain killer shouldn't seem to work quite as well as I had expected.” (14 year old male patient) |
|  |  | “When I'm coughing to use a pillow like squeezes around my chest so the pain doesn't hurt as much” (15 year old male patient). |
|  |  | “I think I just watched a bunch of basketball to keep everything that was going on in the hospital off my mind. Yeah, like just doing other things that I like or playing video games, yeah.” (15 year old male patient). |
|  | ***2.3 Family Dynamics and Perspectives on Surgery and Pain*** | “I would say that the kids that are older and more aware, especially the adolescents, I think that they seem to experience more pain than the younger kids” (Nurse Practitioner/Advanced Practice Nurse) |
|  |  | “I think she thought if she was in pain she wouldn't be able to go home as fast. So she covered it up and said she wasn't.” (Parent of 12 year old patient). |
|  | ***2.4 Self-care activities (i.e. sleep, nutrition)*** | “The last thing I was going to say good sleep during the big kind of get that they're not having sleep derangement before surgery. So that's showing that they can sleep properly, you know kind of have good sleep hygiene going into the surgery. So that afterwards, you know, their sleep hygiene can be kind of returned to normal as soon as possible because sometimes there's a big up and down in that course.” (Physician) |
|  |  | “I think I was really tired and after surgery and with all everything happening, I just wanted to rest and move on to the next day, yeah.” (15 year old male patient). |
|  |  | “Just to get me back to stamina cause I was sleeping for like a long time and in bed for like 3 days straight.” (14 year old female patient). |
|  |  | “... things like, you know, the degree of nutrition or malnutrition plays a role. So they're emaciated and kind of failure to thrive. And I think pain experiences can be worse versus if they're healthier going into their operation.” (Physician). |
|  |  | “But then after I felt a lot better after I got home.” (16 year old male patient) |
|  |  | “... things like, you know, the degree of nutrition or malnutrition plays a role. So they're emaciated and kind of failure to thrive. And I think pain experiences can be worse versus if they're healthier going into their operation.” (Physician) |
| **Category 3: App Feedback** | ***3.1 Goals, Check-ins and Trends*** | “I think the scale of 1 to 5 is definitely better than yes or no, just cause you can like track the amount of pain because you could still be in a little bit of pain or a large amount of pain. It kind of just depends.” (14 year old male patient). |
|  |  | “I felt like the check-in itself though, was like nice. It was pretty intuitive and easy to do, like I did a check-in just when I had the phone. And like, I think it captures like, again, like their ability to move, and their mood, or like big things, sleep is a big thing.” (Physical Therapist). |
|  | - 1. ***Resource Library*** | “I never got any information about how the how I should cope with my anxiety during the time.[…] So I feel like it [app] just say “when was your postponed surgery” and then ask about how or what do you want to cope, what feelings you would you have?” (14 year old female patient) |
|  |  | “Post-acute phase but return to activity is maybe something to do with safe return to school or something, or like maybe safe is not the right word? But, something to do with, like management strategies or return to school.” (Physical Therapist) |
|  |  | “The last thing I was going to say good sleep during the big kind of get that they're not having sleep derangement before surgery. So that's showing that they can sleep properly, you know kind of have good sleep hygiene going into the surgery. So that afterwards, you know, their sleep hygiene can be kind of returned to normal as soon as possible because sometimes there's a big up and down in that course.” (Physician - H12) |
|  | ***3.3 Potential Features & Overall impressions*** | “And a point I just wanted to make is that I see another benefit of using the application. There are a lot of teens who are not very engaged in their own health care, mostly like this is done by the parents. So, this way they can become more engaged. And more knowledgeable. And this would be helpful in the process of transitioning to adult care as well,” (Nurse). |
|  |  | “…as a part of flight plan, make sure you have your stuff so that […] you're gonna be comfortable the day afterwards. You know, things you can do to help with kind of anxiety around it, the kind of pre planning some of the things, you know, things that make you anxious before surgery if possible.” (Physician). |
|  |  | “Well, I think the other piece that does is, you know, we rely so much on our doctors to tell us what everything is all about. But it's so hard to reach them in so many ways, and you know, and then to have something like this I think is super valuable” (Parent of 16 year old male patient) |

**Appendix C: Summary of Proposed App Modifications**

| Feature | Examples |
| --- | --- |
| Goal setting | - To be able to set a goal for a specific time (not just day by day) - Medication management – i.e. being able to set reminders to take pain medication |
| Map out surgical journey | - What to expect of recovery on a day by day basis & when certain activities can be resumed - Considerations for returning to school (i.e. reduce time off, modifications for gym class, infection risk; modifications like rolling bag); 3-sentence summary for talking to friends about time away and health |
| Guided Breathing Exercises | - i.e. Video demo of incentive spirometry with timer - Earn stars for completion |
| Pre-op checklist | - Tool or assessment to identify helpful strategies (i.e. for anxiety management) pre-op so patient has a management plan already in place for post-op - Playlist, packing list/pillow or blanket from home |
| Symptom tracking | - Dizziness, shortness of breath - Tracking pain location and intensity, with option of open text - Potentially update language around mood and worry - Notes list (or prompt to use phone notes to track questions) |
| Notifications | - Include affirmations/friendly language - Default daily time 4pm but customizable in terms of timing and frequency |
| Accessibility features | - Option to listen to library content vs. read |
| Library | - Coping with anxiety – especially pre-operatively, i.e. if surgery is postponed - Distraction activities - Return to activities/school - Recommended Exercise with videos - Chest tubes - Nutrition – suggest food for recovery when you don’t feel like eating - Body image/incision scars - Potential testimonial style information - Searchable - “Education should be tailored enough to be helpful but not too specific that it’s not applicable to everyone” |
| Q&A Chatbot (New feature) |  |
